# Plant extracts have dual mechanism on the protection against dentine erosion: action on the dentine substrate and modification of the salivary pellicle

**DOI:** 10.1038/s41598-023-34256-y

**Published:** 2023-05-01

**Authors:** Samira Helena Niemeyer, Tommy Baumann, Adrian Lussi, Tais Scaramucci, Thiago Saads Carvalho

**Affiliations:** 1 Department of Restorative, Preventive and Pediatric Dentistry, University of Bern, Freiburgstrasse 7, 3010 Bern, Switzerland; 2 Department of Restorative Dentistry, School of Dentistry, University of São Paulo, Av. Prof. Lineu Prestes 2227, Cidade Universitária, São Paulo, SP 05508-000 Brazil

**Keywords:** Dentistry, Preventive dentistry

## Abstract

To investigate the effect of some polyphenol-rich plant extracts on the protection of dentine against demineralization, both acting on the dentine and on the salivary pellicle. Dentine specimens (n = 180) were randomly distributed into 6 experimental groups (n = 30/group): Control (deionized water), Açaí extract, Blueberry extract, Green tea extract, Grape seed extract, and Sn^2+^/F^−^ (mouthrinse containing stannous and fluoride). Each group was further divided into two subgroups (n = 15), according to the site of action of the substance: on the dentine surface (D) or on the salivary pellicle (P). The specimens were submitted to 10 cycles: 30 min incubation in human saliva (P) or only in humid chamber (D), 2 min immersion in experimental substances, 60 min of incubation in saliva (P) or not (D), and 1 min erosive challenge. Dentine surface loss (DSL), amount of degraded collagen (dColl) and total calcium release were analyzed. Green tea, Grape seed and Sn^2+^/F^−^ showed significant protection, with least DSL and dColl. The Sn^2+^/F^−^ showed better protection on D than on P, whereas Green tea and Grape seed showed a dual mode of action, with good results on D, and even better on P. Sn^2+^/F^−^ showed the lowest values of calcium release, not differing only from Grape seed. Sn^2+^/F^−^ is more effective when acting directly on the dentine surface, while Green tea and Grape seed have a dual mode of action: with a positive effect on the dentine surface itself, but an improved efficacy in the presence of the salivary pellicle. We further elucidate the mechanism of action of different active ingredients on dentine erosion, where Sn^2+^/F^−^ acts better on the dentine surface, but plant extracts have a dual mode of action, acting on the dentine itself as well as on the salivary pellicle, improving the protection against acid demineralization.

## Introduction

Frequent contact of tooth surfaces with acids not originated from bacteria results in mineral loss, called dental erosion. With the progression of this condition, enamel is continuously lost and the exposition of the underlying dentine occurs^1^. Epidemiologic studies show a high prevalence of erosive tooth wear worldwide, also regarding severe cases with exposure of dentine already observed in children, adolescents and young adults^2^.

Dentine contains 20% of organic material (mainly collagen), which reduces the amount of dentine loss to a certain degree^3,4^. However, enzymes present in the dentine and in saliva, known as metalloproteinases (MMPs), can degrade the organic matrix, allowing the demineralization to progress^5^. These enzymes are activated at a low pH, such as during an erosive attack, but once the saliva buffers and neutralizes the acids, these activated enzymes can degrade the organic matrix^6^.

Some substances, such as polyphenols, can inhibit the action of MMPs, reducing the degradation of the organic matrix, and slowing the progression of dentine erosion. Polyphenols can inactivate the collagenases by binding to them through hydrogen bonding and hydrophobic interaction, which inhibits collagen degradation^7^. Moreover, polyphenols act as collagen cross-linkers, increasing the resistance of the collagen to degradation^8^, showing a dual action on dentine protection against erosion. The most studied MMP inhibitor is green tea, whose effect is related to the presence of polyphenols, especially the epigallocatechin gallate (EGCG).

The progression of dental erosion is also controlled by the acquired salivary pellicle, which forms by the adsorption of proteins, peptides, lipids and other macromolecules present in saliva to the tooth surface^9^. The pellicle protects the tooth from direct contact with the erosive acids; however, this protection is limited, especially under severe erosive challenges^10^. Most studies on the protective effect of the pellicle are on enamel, and only a few studies have been published on dentine, so more studies on this topic are necessary^11^.

To counteract the limited protection from the pellicle, this organic layer can be modified for enhanced protection. This has already been tested for the protection of enamel against dental erosion^12–18^, however little is known about the effect of pellicle modification on dentine. One possibility of pellicle modification is the application of plant extracts that are rich in polyphenols, which in turn have affinity for proteins^9,14^. They act by inducing precipitation and aggregation of salivary proteins, which will adsorb to the pellicle surface^19^. Cross-linking of salivary proteins by polyphenols results in thicker pellicle layers that are more resistant to removal^14,20^.

Plant extracts rich in polyphenols have promising potential to protect dentine against erosion, since they will potentially have a dual action: they can bind to salivary proteins and modify the salivary pellicle, as well as bind to collagen in dentine and increase its resistance to degradation. However, up to the present moment, no study has tested this possible dual action. Therefore, the present study investigated the effect of some polyphenol-rich plant extracts on the protection of dentine against demineralization, both acting on the dentine and on the salivary pellicle. The specific aims of the present study were: (1) to verify if the plant extracts have a direct effect on the dentine, causing less dentine loss; (2) to verify if plant extracts can modify the salivary pellicle, improving its protective effect against dentine erosion; (3) to analyze the amount of degraded collagen after collagenase treatment. The null hypotheses were: (1) the plant extracts do not have a direct effect on the dentine, protecting it against erosion; (2) the plant extracts do not modify the salivary pellicle, improving its protective effect; (3) the plant extracts do not reduce the amount of collagen degradation.

## Material and methods

### Dentine specimen preparation

A total of 180 dentine fragments were obtained from the cervical area of the root of extracted human premolars. The teeth had been taken from a pooled biobank (details under Ethical Aspects). The fragments were embedded in acrylic resin and these specimens were then ground flat with abrasive silicon carbide paper discs of grain size 18 µm until 300 µm of the root surface was removed. The specimens were further ground with abrasive silicon carbide paper discs of 9 µm grain size to remove another 30 µm of the dentine surface. Final polish was performed with the paper discs with grain size of 3 µm for 2 min. Grinding and polishing procedures were performed with a polishing machine under constant cooling (EXAKT-Apparatebau, Otto Herrman, Nortedstedt). Between each type of paper disc and after the final polish, the specimens were rinsed and sonicated for 1 min. The specimens were stored in tap water until the beginning of the experiment when they were rinsed and sonicated for 1 min. Then, the initial profile of a central area (3 mm × 1 mm) was analyzed with an optical profilometer, and an adhesive tape was placed on two parts of the dentine surface, with a distance of 1.5 mm between them, where the treatments and challenges were performed. The specimens were kept in a humid chamber during the whole experiment.

### Stimulated whole human saliva collection

Healthy adults, who agreed to donate saliva, chewed on a paraffin wax for 10 min and all the stimulated saliva was collected in chilled vials. The saliva collected from 15 donors was immediately pooled. Then, the saliva pool was centrifuged (20 min; 4 °C; 4,000g) and the supernatant was distributed in small aliquots, which were stored at − 80 °C until use. This procedure was performed in the morning and the volunteers were asked not to eat or drink anything, except for water, for 2 h before the saliva collection^21^.

### Ethical aspects

The study was carried out in accordance with approved guidelines and regulations of the local Ethics Committee (Kantonale Ethickkomission, KEK), where pooled samples of teeth and saliva are categorized as “irreversibly anonymized” and therefore exempt of ethical approval. The volunteers were previously informed about the use of their teeth and/or their saliva in research and their informed consent was obtained. Since the teeth and saliva used in the present study were pooled, according to KEK previous ethical approval is not required.

### Experimental groups

The specimens were randomly distributed into 6 main experimental groups (n = 30/group), according to the test substances. Four groups were plant extracts: Açaí extract (Bio-Açaí Pulver, ZeinPharma, Germany; 33 g polyphenol per 100 g powder), Blueberry extract (Heidelbeer-Extrakt mit Anthocyanen, Fairvital B.V., Germany; 125 mg polyphenol per capsule), Green tea extract (Green Tea Deluxe, ZeinPharma, Germany; 250 mg polyphenol, namely EGCG, per capsule), and Grape seed extract (OPC, Fairvital B.V., Germany; 300 mg polyphenol, namely OPC, per capsule). As negative control (Control), deionized water was used. As positive control, a commercial stannous- and fluoride-containing mouthrinse (Sn^2+^/F^−^) was used (elmex Erosionsschutz, GABA, Switzerland, pH 4.5). The solutions containing plant extracts were prepared daily, with a polyphenol concentration of 2 g/l, according to the total polyphenol content declared by the manufacturer. The pH of the solutions containing the plant extracts was adjusted to 5.2, but the pH of the water and the mouthrinse were not adjusted.

To test the dual action of the solutions, each group was further divided into two subgroups (n = 15), where the solutions were either applied directly onto the dentine surface (D) or they were used to modify the salivary pellicle (P) formed on the specimen.

### Experimental procedures

For the subgroup with salivary pellicle formation (P), the specimens were initially incubated with clarified whole human saliva for 30 min at 37 °C. Modification of the pellicle was carried out with the different solutions by immersing the specimens for 2 min (25 °C, 70 rpm, travel path 50 mm) in each solution, according to the group. Then, the specimens were again incubated in human saliva for a further 60 min at 37 °C. After each step, the specimens were washed in deionized water and the excess water removed with an absorbent paper, without touching the experimental area.

The specimens where the solutions were applied directly onto the dentine surface (D) were treated similarly, but not exposed to saliva. They were placed in a humid chamber for 30 min at 37 °C, then they were exposed directly to the test substances, as described above (immersion for 2 min, 25 °C, 70 rpm, travel path 50 mm), and again placed in a humid chamber for a further 60 min at 37 °C. The specimens were washed as described before.

Afterwards, all specimens were individually submitted to an erosive challenge (1 min, 1% citric acid, 25 °C, 70 rpm, travel path 50 mm), washed with deionized water and the excess water was removed with an absorbent paper, without touching the experimental area. The citric acid was stored for calcium analysis (CaR), and the specimens were again submitted to the same cyclic procedure. The sequence of pellicle/no pellicle formation, incubation in the test substances, further pellicle/no pellicle formation, and the erosive challenge was repeated 10 times. After 10 cycles, the specimens were submitted to dentine surface loss (DSL) measurements.

### Determination of the amount of calcium released to the citric acid (CaR)

From the citric acid used for each specimen, an aliquot of 1 ml from each cycle was taken, totaling 10 ml of acid. This 10 ml aliquot was mixed with 0.5% lanthanum nitrate (La(NO_3_)_3_·6H_2_O), and then the total calcium concentration in the solution was analyzed with an atomic absorption spectrometer (AAS; AAnalyst 400, Perkin Elmer Analytical Instruments, Waltham, MA, USA). The measured amount of calcium was normalized to the exposed dentine surface area, and the results are presented as nmol calcium/mm^2^ dentine. To determine the dentine exposed area, a light microscope connected to a camera (Leica, M420 and Leica, DFC495, respectively) under 20 × magnification and the software program IM500 were used. The contour of the exposed dentine area was traced by an experienced operator and the area was calculated with the specific software program. The total amount of calcium was determined considering all 10 erosive cycles for each specimen individually.

### Dentine surface loss (DSL) measurements

During specimen preparation, a central area (3 mm x 1 mm) of the dentine had been selected for analyses. This area includes one uncovered area in the middle of the dentine fragment (1.5 mm x 1 mm) exposed to the experimental procedures, and two covered areas on either side of the exposed area (each measuring 0.75 mm x 1 mm) serving as references.

All specimens were scanned with an optical profilometer (MicroProf 100, FRT the art of Metrology, Germany), while the dentine moisture was controlled to avoid shrinkage of the organic matrix, by adding a drop of deionized water on the dentine surfaces for 30 s before the analysis. The excess of water was removed with an absorbent paper, without touching the analyzed surface, right before the initial of the measurements. The same 3 mm x 1 mm central area was scanned at three experimental time points: initially (after specimen preparation), immediately after 10 experimental cycles (dSL10cycles) and after removal of the demineralized organic matrix (dSLcollagenase). For the latter, the specimens were incubated in a mineral solution (4.08 mM Na_3_PO_4_, 20.10 mM KCL, 11.90 mM Na_2_CO_3_, 1.98 mM CaCl_2_; pH 6.5) containing collagenase from Clostridium histolyticum type VII (100 units/ml), for 65 h at 37 °C.

All scans were normalized to the initial surface curvature of the specimens, by subtracting the initial scan (after specimen preparation) from the final scan (dSLcollagenase). After normalization, dentine surface loss (DSL) was determined by subtracting the mean height of the exposed area from the mean height of both reference areas considering the last scan after removal of the demineralized organic matrix (dSLcollagenase). Lastly, the height of the dentine collagen layer (dColl) was determined for each specimen by calculating the difference in height before (dSL10cycles) and after (dSLcollagenase) removal of the demineralized organic matrix (dColl = dSLcollgenase—dSL10cycles). All analyses were made using the specific software (FRT Mark III, FRT the art of Metrology, Germany) of the optical profilometer.

### Statistical analysis

Data were evaluated with Shapiro–Wilk normality test and due to lack of normal distribution of some groups, non-parametric tests were used. Kruskal–Wallis for comparison between groups, considering each subgroup separately, was performed for each response variable: CaR, DSL and dColl. Post hoc Dunn’s test was used, and the p-value corrected for multiple comparisons was considered. To assess the dual effect of the solutions acting directly on dentine or on the pellicle, each group was then analyzed with Mann–Whitney U test comparing the subgroups D and P. Significance level was set at 5% and the analyses were performed with GraphPad Prism 7 for Mac.

### Statement of ethics

This study is reported according to the COPE guidelines.

## Results

**Figure 1:**
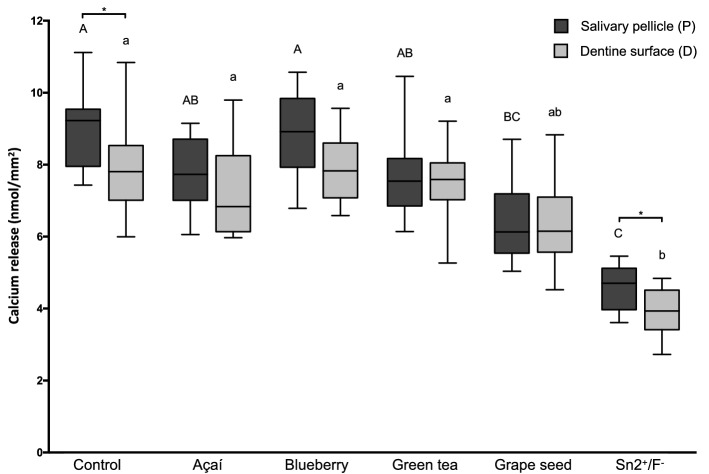
Boxplots of the total amount of calcium released to the citric acid per mm^2^ of dentine. Different lower-case letters denote significant differences between the experimental groups for the action directly on the dentine surface (D). Different upper-case letters denote significant differences between the experimental groups for the action on the salivary pellicle (P). Bar with asterisk shows significant difference between D and P within the same group.

The amount of calcium released (median nmol/mm^2^, interquartile range) by the specimens to the citric acid is presented in Fig. 1. When acting directly on the dentine, only Sn^2+^/F^−^ (3.94, 3.41–4.51) provided protection, releasing significantly less calcium than the control group (7.81, 7.01–8.53; p < 0.0001). When acting in the presence of the salivary pellicle, both Grape seed (6.13, 5.54–7.19) and Sn^2+^/F^−^ (4.70, 3.97–5.12) showed protective effect, with lower amounts of calcium release than in the Control group (9.23, 7.95–9.54; p = 0.0012 and p < 0.0001, respectively). Comparing the corresponding subgroups treated with the same test substances, only the Control (D: 7.81, 7.01–8.53; P: 9.23, 7.95–9.54; p = 0.0041) and Sn^2+^/F^−^ (D: 3.94, 3.41–4.51; P: 4.70; 3.97–5.12; p = 0.0078) groups showed significant differences, where the presence of the pellicle caused higher calcium release.

**Figure 2:**
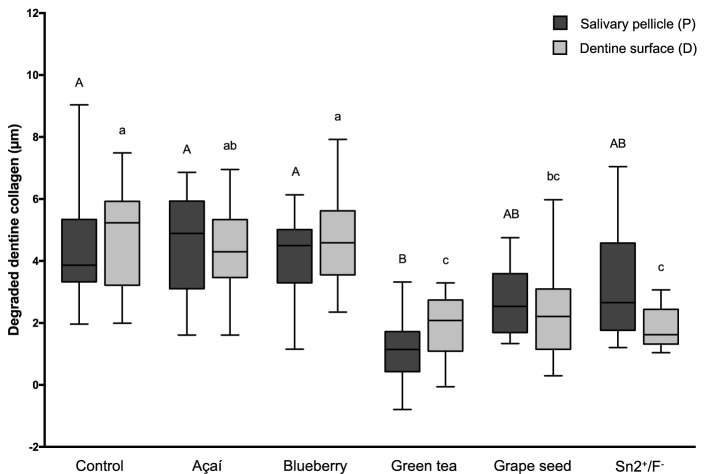
Boxplots of the degraded dentine collagen (dColl). Different lower-case letters denote significant differences between the experimental groups for the action directly on the dentine surface (D). Different upper-case letters denote significant differences between the experimental groups for the action on the salivary pellicle (P). Bar with asterisk shows significant difference between D and P within the same group.

The height of the collagen layer (dColl; median µm, interquartile range) is presented in Fig. 2. When acting directly on the dentine, Green tea (2.08, 1.09–2.74), Grape seed (2.21, 1.15–3.10) and Sn^2+^/F^−^ (1.62, 1.32–2.44) showed lower amounts of collagen, significantly different from the control group (5.23, 3.22–5.92; p = 0.0009, p = 0.0116, p = 0.0005, respectively). However, when acting on the salivary pellicle, only Green tea (1.15, 0.43–1.72) significantly differed from the control group (3.86, 3.33–5.34), presenting significantly lower amounts of collagen (p < 0.0001). Considering the site of action of the solutions, there was no difference in the height of the collagen layer when acting directly on the dentine surface or when the salivary pellicle was present for any of the experimental groups.

**Figure 3:**
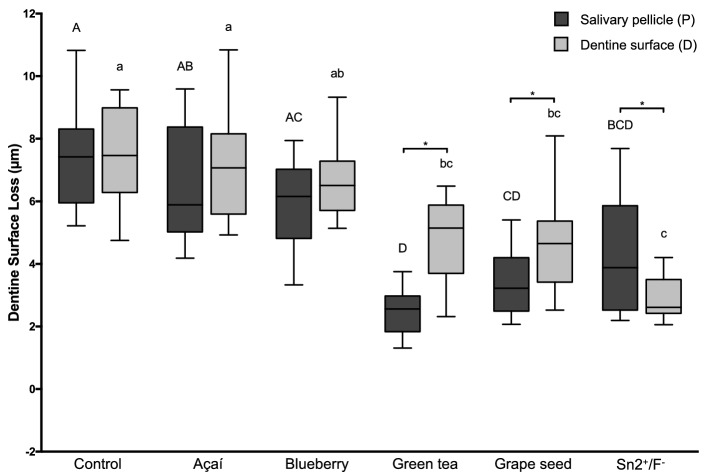
Boxplots of dentine surface loss. Different lower-case letters denote significant differences between the experimental groups for the action directly on the dentine surface (D). Different upper-case letters denote significant differences between the experimental groups for the action on the salivary pellicle (P). Bar with asterisk shows significant difference between D and P within the same group.

**Figure 4:**
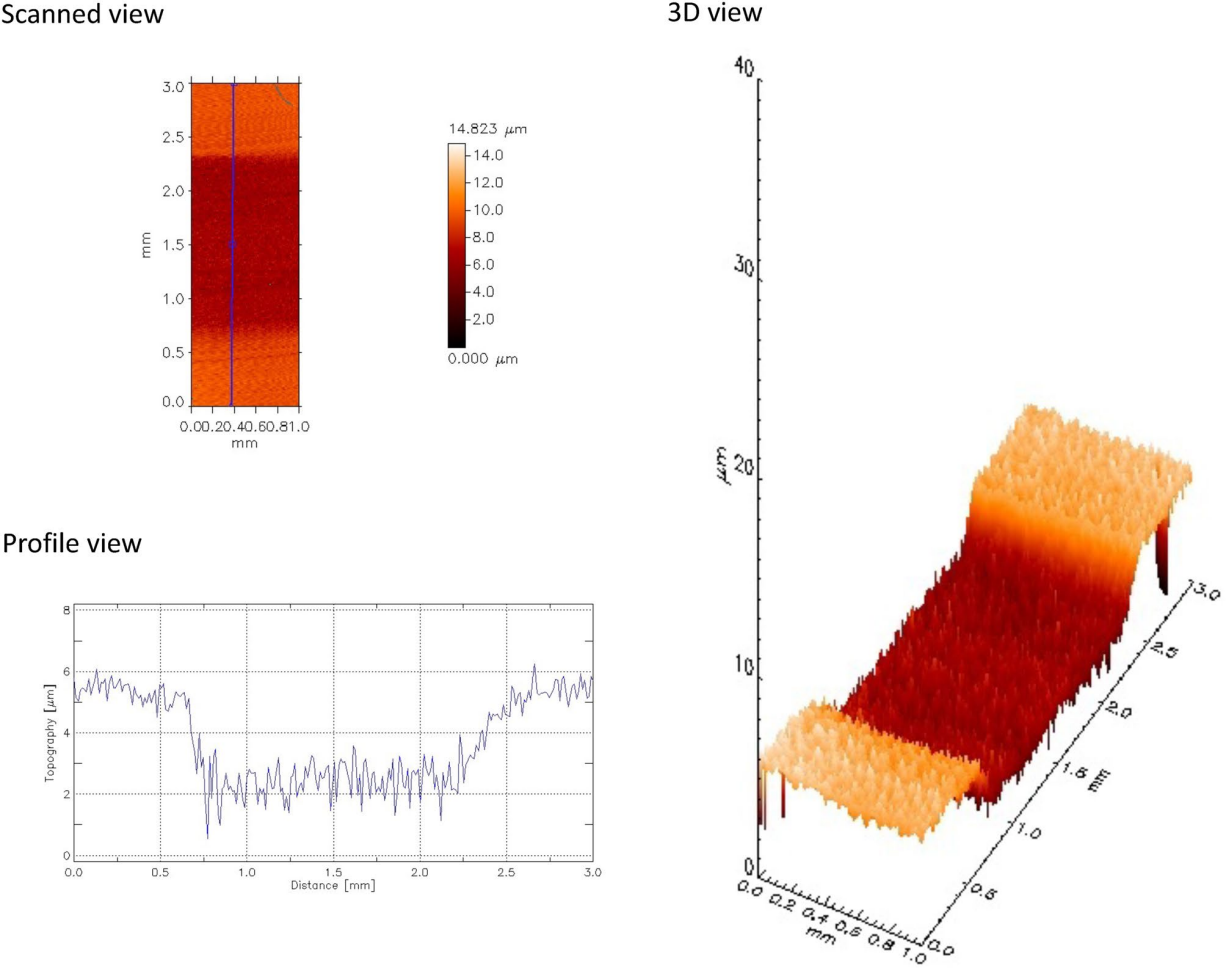
Representative images of dentine surface loss obtained with the optical profilometer after 10 cycles.

The total dentine surface loss (DSL; median µm, interquartile range) is shown in Fig. 3. We observe a similar pattern as in dColl. When the solutions acted directly on the dentine surface, Green tea (5.15, 3.70–5.88), Grape seed (4.65, 3.42–5.37) and Sn^2+^/F^−^ (2.61, 2.42–3.50) significantly protected against demineralization, and were different from the control group (7.47, 6.28–8.99; p = 0.0090, p = 0.0031 and p < 0.0001, respectively). When the salivary pellicle was present, these same groups showed a similar protection, with significantly lower DSL values than the control group (Green tea: 2.56, 1.83–2.98, p < 0.0001; Grape seed: 3.23, 2.50–4.20, p = 0.0001; Sn^2+^/F^−^: 3.88, 2.53–5.86, p = 0.0063; Control: 7.42, 5.95–8.31). However, when comparing the two sites of action of each treatment group (directly on the dentine surface or on the salivary pellicle), we observed significant differences for Green tea (p < 0.0001), Grape seed (p = 0.0256) and Sn^2+^/F^−^ (p = 0.0469). While Green tea (D: 5.15, 3.70–5.88; P: 2.56, 1.83–2.98) and Grape seed (D: 4.65, 3.42–5.37; P: 3.23, 2.50–4.20) showed significantly better protection when the salivary pellicle was present, Sn^2+^/F^−^ (D: 2.61, 2.42–3.50; P: 3.88, 2.53–5.86) showed better protection when acting directly on the dentine surface. Figure 4 shows representative images (scanned, profile and 3D view) of dentine surface loss obtained with the optical profilometer after 10 cycles.

## Discussion

It is well established that the erosion process on dentine is particularly affected by the layer of the organic matrix. With increasing acid attacks, the mineral component of dentine is readily demineralized, and the organic component is exposed. The amount of exposed organic layer increases as the erosion process progresses, but when this layer reaches a certain thickness, it acts as barrier against further acid attacks, and the demineralization is slowed down^3,4^. In the presence of saliva, the organic layer can be degraded by salivary enzymes, allowing the erosion process to advance further^5^. The use of human saliva in our experimental design, however, did not greatly affect the organic layer. This was observed in the lack of difference in the amount of degraded collagen between the subgroups of dentin surface (D) and salivary pellicle (P) within the control group (Fig. 2). The erosive process used in the present study can be considered mild, because we only used a total of 10 min of acid challenges.

As expected, we observed a protective effect from Green tea and Grape seed extracts and from Sn^2+^/F^−^. But interestingly, Green tea and Grape seed showed significantly better protection when the salivary pellicle was present, whereas Sn^2+^/F^−^ showed better protection acting directly on the dentine surface. The protection from the commercial mouthrinse is mostly related to the fluoride and stannous ions, which can form an acid resistant layer on dental surfaces, thereby preventing the direct contact of the acids, and slowing the rate of demineralization^21^. On dentine, the mode of action of stannous ions is influenced by the layer of organic matrix^22^. If the organic layer is constantly removed by enzymes, the stannous ions can form precipitates on the dentine surface; however, if the organic layer is maintained (as in the specimens of our study) then its action is rather related to a diffusion-controlled incorporation of stannous ions into the underlying dentine^22,23^. Remarkably, our results now show that the presence of the salivary pellicle will influence the efficacy of Sn^2+^/F^−^ mouthrinse. When the salivary pellicle was present, the solution containing stannous and fluoride showed significantly less protection than when acting directly on the dentine surface. We speculate that the presence of the salivary pellicle can hinder, to some degree, the incorporation of stannous ions into the dentine. Despite of this worsened protective effect in the presence of saliva, the stannous solution still showed better protection than the Control group, which is in accordance with other studies^24,25^. Although some studies have pointed out the possibility of stannous to cross-link salivary proteins in the pellicle^16,26^, our results point to a weaker protective effect on the pellicle, and the stannous and fluoride solution will have a greater effect directly on the dentine surface. Furthermore, the stannous solution might also have an additional protective effect on the organic matrix, where it inhibits the enzymes in saliva^27^.

In the present study, we also observed a protective effect for Green tea and Grape seed extracts, which, remarkably, provided better protection when the salivary pellicle was present. This suggests that these agents were able to modify the salivary pellicle, increasing its acid resistance, so our second null hypothesis was rejected. These extracts, especially the Green tea, also showed the lowest amount of collagen degradation, which allows for the rejection of the first and third null hypotheses.

Green tea is obtained from Camelia sinensis leaves and it has been shown to protect dentine against demineralization^28,29^. This is mainly due to the effect of epigallocatechin-3-gallate (EGCG)^30^, which contains galloyl moieties that play an important role in protecting against dentine degradation. The galloyl moiety can inhibit proteases by changing their conformation, therefore hindering their binding to collagen^31^. Besides, the EGCG is able to chelate the zinc in the MMPs, which inhibits the MMPs’ effect on collagen degradation^7,30,32^. Since our study showed no degrading effect from the enzymes in saliva, it is likely that EGCG acted in the collagen layer in our specimens. The galloyl moiety in EGCG contains many hydroxyl groups, which can form more hydrogen bonds with the collagen in the organic layer. This, in turn, improves the dentine mechanical properties, reduces collagen degradation^31^, and reduces erosive demineralization of the dentine. The Green tea extract solution used in the present study contains 2 g/l of EGCG, which explains the great reduction of collagen degradation observed for this group. The ability of EGCG to reduce collagen degradation of eroded dentine has been already observed^8,33^. In the presence of a salivary pellicle, Green tea was able to further reduce DSL, implying that it can successfully modify the salivary pellicle. Similar to the collagen in the organic layer of dentine, the galloyl groups in EGCG also have high binding affinities to the salivary proteins, so they can bind to the salivary pellicle^34^, increasing its thickness and its protection against acid challenges^9^. This causes an additive effect, where the Green tea has a positive effect on the dentine itself, but when the pellicle is present, its effect on the proteins significantly increases the protective effect, supporting the dual mode of action of Green tea. Interestingly, despite the positive results observed on surface loss and on height of collagen layer, the positive effect of Green tea was not detected by the calcium release analysis, and more investigations are still necessary to elucidate this fact. Furthermore, Green tea is known to contain fluoride, but it is unlikely that this had considerable impact on its protective effect because the fluoride concentration is too low^29^, and the protective effect of fluoride against dentine erosion is limited^21^.

Likewise, Grape seed extract was able to protect the dentine against surface loss, and this effect was more pronounced in the presence of the salivary pellicle. In a similar manner to Green tea, the polyphenols of the grape seed extract can bind to the organic layer in the dentine itself, and also to the proteins in the salivary pellicle, thereby increasing its thickness and acid resistance^13,14^. Regarding its effect on the organic layer of the dentine, our results are in agreement with other studies^8,35^. Grape seed extract contains oligomeric proanthocyanidins, which are known as a natural cross-linking agent. In dentine, the proanthocyanidins are able to cross-link collagen^36^, improving the mechanical properties of the dentine surface. Regarding the presence of salivary pellicle, our study showed a significantly better effect than when acting only on the dentine surface. This indicates that Grape seed extract also demonstrates a dual mode of action, interacting with the organic layer of dentine, as well as modifying the salivary pellicle formed on the dentine surface. This is an interesting result, because the protective effect of Grape seed extract on the pellicle had already been observed for pellicles formed on enamel^14^, but not yet for pellicles formed on dentine. Also, although there was no difference between Grape seed extract and the Control group in the calcium analysis when the solutions acted directly on the dentine, the protection from Grape seed extract was observed when the salivary pellicle was present. This again shows its positive effect when cross-linking pellicle proteins.

Although Açai and Blueberry are rich in polyphenols, they unexpectedly did not produce the positive results observed for Green tea or for Grape seed extract. The lack of protective effect observed for these groups might be related to the type or configuration of polyphenols present. Açai and Blueberry contain polyphenols of the group of anthocyanins. Açai contains mainly cyanidin 3-glucoside and cyanidin 3-rutinoside, whereas Blueberry contains malvidin-3-glucoside and malvidin-3-galactoside, which probably have lower binding affinities to the proteins in the salivary pellicle or to collagen. The number of hydroxyl groups could also have an influence here, of which EGCG has more than cyanidin or malvidin. More hydrogen bonds are therefore possible with EGCG, which could lead to more and stronger interactions with proteins. Therefore, it could be that those Blueberry and Açai polyphenols could have better results if applied at higher concentrations, but further investigations are necessary to better understand the interaction of these polyphenols and these structures. Still, our results suggest that not all polyphenols will be able to protect against dental erosion, even though they might marginally modify the pellicle^37^ or bind to collagen.

When observing the two subgroups of the Control group (D and P), we observed no difference in dentine surface loss. This can be explained by the limited protection of the salivary pellicle on dentine^10,38^. Regarding the higher CaR observed in subgroup P, we can speculate that this was due to calcium released from the salivary pellicle itself, in addition to that of the demineralized dentine. In contrast, the groups treated with the plant extracts did not show differences in CaR between the subgroups D and P. We can suppose that the polyphenols in these groups were able to bind to and to modify the pellicle, turning it denser and more resistant to the citric acid^13^. Therefore, the calcium from the pellicle could not be as easily released as in the other groups.

Our results show that some polyphenols rich substances seem to have a dual mode of action on dentine. Previously, most studies focused on the mode of action directly on the dentine itself, emphasizing the effect of the polyphenols on the collagen layer. We now show that these products also have an effect on the salivary pellicle, where they produce even better protective results than when acting purely on the dentine surface. In contrast, the stannous-containing solution has a better protective effect on the dentine surface, and the presence of the salivary pellicle is slightly hindering. This implies that further studies should be similarly made regarding other products, to verify their mode of action when the pellicle is present. Furthermore, considering the possible complementary approach of the natural plant extracts and the stannous and fluoride ions, it would be worth testing the association of these agents. It is important to point out that although we have used human saliva, the salivary pellicle was formed in vitro, and in situ studies would be necessary to further elucidate the effect of plant extracts on salivary pellicle modification. Future studies should also investigate the impact of different substances on the proteomic profile of the pellicle, and whether polyphenols alter the activities of salivary and dentin proteases.

We conclude that the Green tea extract, Grape seed extract and Sn^2+^/F^−^ groups were able to significantly protect the dentine against erosion. While Sn^2+^/F^−^ mainly acts on the dentine surface itself, with the salivary pellicle slightly hindering its effect; the Green tea and Grape seed extracts showed a dual mode of action, acting directly on the dentine itself, but even more effectively in the presence of the salivary pellicle.

## Data Availability

All data generated or analyzed during this study are included in this article. Further enquiries can be directed to the corresponding author.
